# Distinct clinical characteristics and helminth co-infections in adult tuberculosis patients from urban compared to rural Tanzania

**DOI:** 10.1186/s40249-018-0404-9

**Published:** 2018-03-24

**Authors:** George Sikalengo, Jerry Hella, Francis Mhimbira, Liliana K. Rutaihwa, Farida Bani, Robert Ndege, Mohamed Sasamalo, Lujeko Kamwela, Khadija Said, Grace Mhalu, Yeromin Mlacha, Christoph Hatz, Stefanie Knopp, Sébastien Gagneux, Klaus Reither, Jürg Utzinger, Marcel Tanner, Emilio Letang, Maja Weisser, Lukas Fenner

**Affiliations:** 1 Ifakara Health Institute, Dar es Salaam, Tanzania; 2 Swiss Tropical and Public Health Institute, Basel, Switzerland; 3 University of Basel, Basel, Switzerland; 4 Institute of Social and Preventive Medicine, University of Bern, Bern, Switzerland

**Keywords:** Co-infection, Helminth infection, Recurrent tuberculosis, Schistosomiasis, Tanzania, Tuberculosis

## Abstract

**Background:**

Differences in rural and urban settings could account for distinct characteristics in the epidemiology of tuberculosis (TB). We comparatively studied epidemiological features of TB and helminth co-infections in adult patients from rural and urban settings of Tanzania.

**Methods:**

Adult patients (≥ 18 years) with microbiologically confirmed pulmonary TB were consecutively enrolled into two cohorts in Dar es Salaam, with ~ 4.4 million inhabitants (urban), and Ifakara in the sparsely populated Kilombero District with ~ 400 000 inhabitants (rural). Clinical data were obtained at recruitment. Stool and urine samples were subjected to diagnose helminthiases using Kato-Katz, Baermann, urine filtration, and circulating cathodic antigen tests. Differences between groups were assessed by *χ*^2^, Fisher’s exact, and Wilcoxon rank sum tests. Logistic regression models were used to determine associations.

**Results:**

Between August 2015 and February 2017, 668 patients were enrolled, 460 (68.9%) at the urban and 208 (31.1%) at the rural site. Median patient age was 35 years (interquartile range [IQR]: 27–41.5 years), and 454 (68%) were males. Patients from the rural setting were older (median age 37 years vs. 34 years, *P* = 0.003), had a lower median body mass index (17.5 kg/m^2^ vs. 18.5 kg/m^2^, *P* <  0.001), a higher proportion of recurrent TB cases (9% vs. 1%, *P* <  0.001), and in HIV/TB co-infected patients a lower median CD4 cell counts (147 cells/μl vs. 249 cells/μl, *P* = 0.02) compared to those from urban Tanzania. There was no significant difference in frequencies of HIV infection, diabetes mellitus, and haemoglobin concentration levels between the two settings. The overall prevalence of helminth co-infections was 22.9% (95% confidence interval [*CI*]: 20.4–27.0%). The significantly higher prevalence of helminth infections at the urban site (25.7% vs. 17.3%, *P* = 0.018) was predominantly driven by *Strongyloides stercoralis* (17.0% vs. 4.8%, *P* <  0.001) and *Schistosoma mansoni* infection (4.1% vs. 16.4%, *P* <  0.001). Recurrent TB was associated with living in a rural setting (adjusted odds ratio [a*OR*]: 3.97, 95% *CI*: 1.16–13.67) and increasing age (a*OR*: 1.06, 95% *CI*: 1.02–1.10).

**Conclusions:**

Clinical characteristics and helminth co-infections pattern differ in TB patients in urban and rural Tanzania. The differences underline the need for setting-specific, tailored public health interventions to improve clinical management of TB and comorbidities.

**Electronic supplementary material:**

The online version of this article (10.1186/s40249-018-0404-9) contains supplementary material, which is available to authorized users.

## Multilingual abstracts

Please see Additional file 1 for translations of the abstract into the five official working languages of the United Nations.

## Background

Worldwide, tuberculosis (TB) is the leading cause of mortality from an infectious disease surpassing human immunodeficiency virus (HIV) infection [1]. Globally, the burden of TB is decreasing, but mortality due to TB remains high with 1.4 million TB deaths and an estimated 10.4 million new cases in 2015 [1]. The global TB case detection rate is below 63% and even lower in Tanzania with a detection rate ranging from 42% to 54% [2, 3]. This is partly due to frequent delays in TB diagnosis in low-income settings [4–8] ranging from 25 to 185 days [5–7]. Delay in TB diagnosis is associated with increased transmission in the community [9]. A deeper understanding of the epidemiology of TB is needed in order to reach the ambitious vision of the End TB strategy of zero TB discrimination, disease suffering, and deaths by 2035 [10, 11].

In Tanzania the prevalence of TB varies considerably across regions, and is higher among males, older persons, and those with lower socioeconomic status [2]. Studies have shown different epidemiological features of TB in urban and rural settings due to differences in health-seeking behaviour, knowledge of TB transmission, gender roles, socioeconomic status, and disease burden [12–14]. Rural-urban characteristics and living conditions could account for differences in the epidemiology of TB in Tanzania and elsewhere. Comorbidities such as HIV and helminth co-infections contribute to different treatment outcomes among TB patients [15–17]. We studied differences in the epidemiology of TB and comorbidities such as helminth co-infections and severe anaemia from two population-based TB cohort platforms established since 2013 in Tanzania [18].

## Methods

### Study setting

**Fig. 1:**
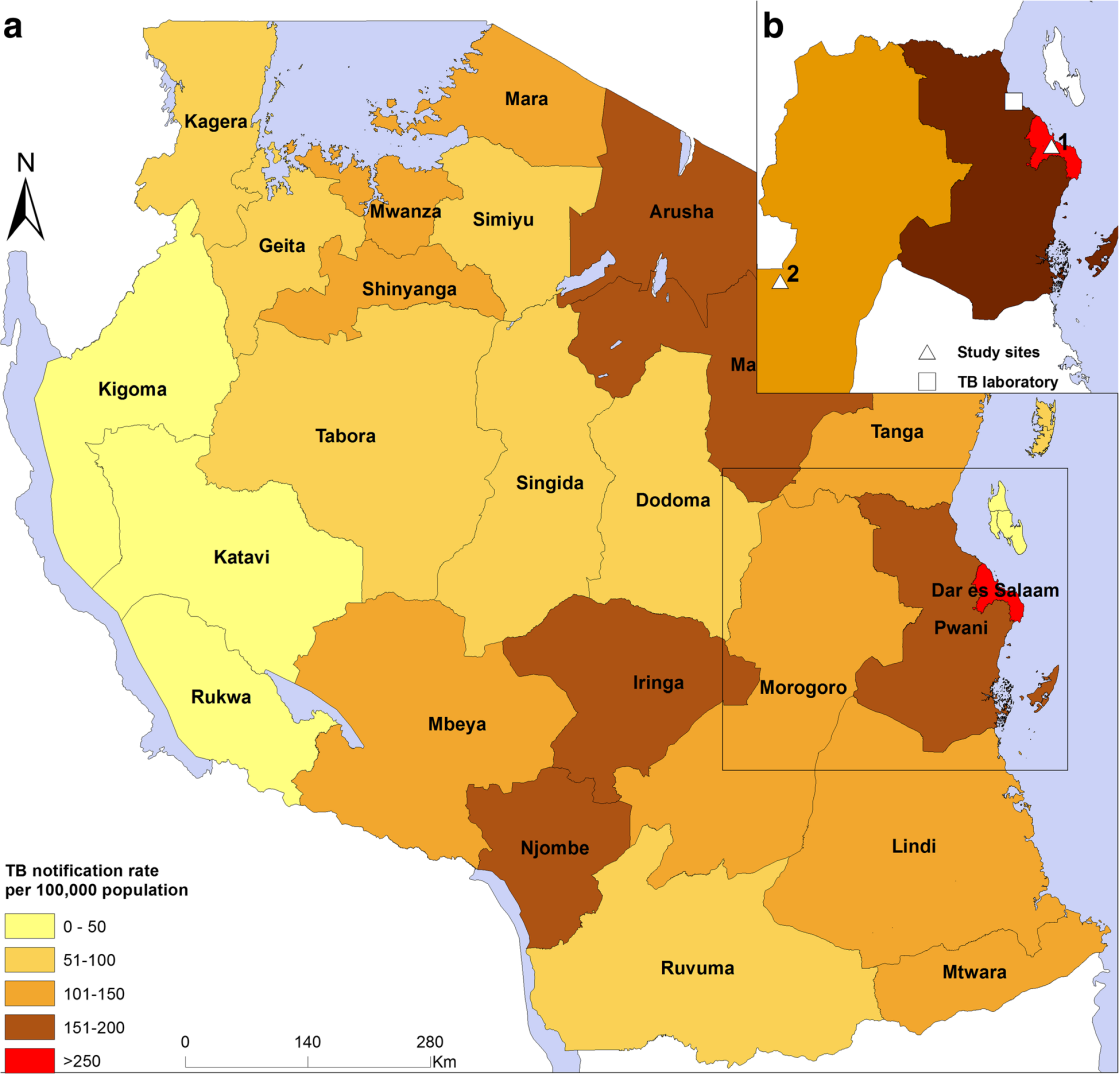
Map of Tanzania showing the regional tuberculosis (TB) notification rates, the locations of the study sites (triangles), and the TB laboratory (square). **a** Overview. **b** Study site 1 (urban), Temeke District, Dar es Salaam Region (triangle); study site 2 (rural), Ifakara, Kilombero District (triangle); and the tuberculosis laboratory in Bagamoyo, Pwani Region

We included adult patients (≥ 18 years) from an ongoing prospective cohort of bacteriologically confirmed pulmonary TB patients in Tanzania (TB-DAR). TB-DAR was initiated in 2013 as a platform to study clinical and molecular epidemiology of TB in Tanzania. The study has two recruitment sites (Fig. 1): one urban site in the densely populated Temeke district in Tanzania’s economic capital, Dar es Salaam, with ~ 4.4 million inhabitants, and one in the rural site found within the Ifakara ward in the sparsely populated Kilombero district with ~ 400 000 inhabitants [19].

### Study sites

#### Urban site (Dar es Salaam)

The urban site is located in the Temeke District in Dar es Salaam inhabited by about 1.4 million inhabitants (Fig. 1). Patient recruitment was done at the TB clinic of Temeke District Hospital, which is one of three regional referral hospitals in the city. The hospital is the largest healthcare facility in the district that provides specialized care and treatment for the Temeke population. Recruitment of patients started in November 2013 and is ongoing in the second part of 2017 [18, 20–22].

#### Rural site (Ifakara)

The rural site is located in the Kilombero District with a population of about 407 000 people [19]. Recruitment was done at the Chronic Disease Clinic of Ifakara (CDCI), a clinic for patients infected with HIV and/or TB at the Saint Francis Referral Hospital (Fig. 1). The hospital is the largest healthcare facility of the Kilombero District in the Morogoro Region, located in southern Tanzania, and provides care for residents of Kilombero and the nearby Ulanga District [23]. Patient recruitment started in August 2015 and is ongoing at the time of manuscript writing.

### Study population

**Fig. 2:**
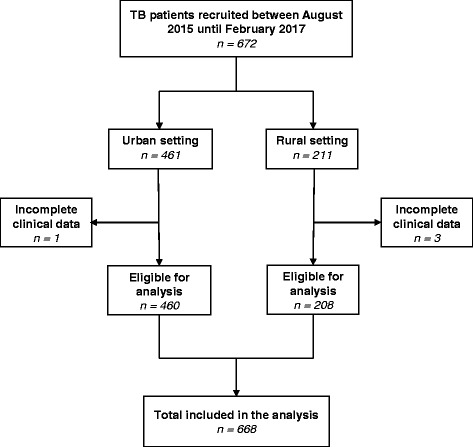
Selection of study patients

We analysed data collected from patients who were consecutively enrolled between August 1, 2015 and February 28, 2017. Patients were eligible for enrollment if they had an age of 18 years or above, lived within the study area, and gave written informed consent. Patients who were severely ill were excluded. Of the 672 bacteriologically confirmed TB patients enrolled in the TB-DAR study during this time period, we excluded four patients due to missing complete clinical information (Fig. 2).

**Fig. 3:**
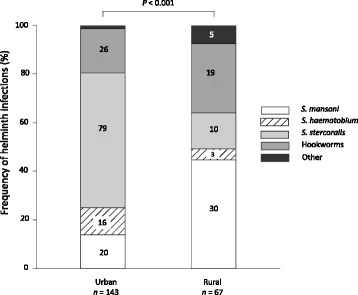
Frequency distribution of helminth species among adult TB patients co-infected with any helminth in an urban and rural setting in Tanzania (in percentage). Numbers on bars represent absolute numbers, the category “Other” includes *Ascaris lumbricoides* and *Trichuris trichiura*. All positive helminth results were analysed (including results from patients with multiple helminth infection)

### Study procedures

At recruitment, patients with confirmed pulmonary TB were interviewed and underwent physical examination as previously described [18, 22]. After recruitment, patients were seen by the study doctor at 6 and 12 months after initiating TB treatment. Of note, TB treatment was supervised by home-based or facility-based direct observation (patient-centered approach) according to the National guideline [24]. Clinical data and biological specimens (sputum, serum, plasma, stool, and urine samples) were collected from all TB patients for laboratory analysis.

### Laboratory procedures

#### Microbiological investigations

Bacteriological confirmation of TB was done by examining the presence of acid fast bacilli (AFB) under fluorescence LED microscope or by using Xpert MTB/RIF assay (Cepheid; Sunnyvale, USA) in all sputum samples. AFB smear-positive results were graded according to published guidelines [24, 25]. All sputum samples were sent to a biosafety level 2+ TB laboratory for solid culture on Lowenstein-Jensen medium at the Bagamoyo Research and Training Center (BRTC), Bagamoyo, Tanzania (Fig. 1). Sputum samples from Ifakara were preserved in cetylpyridinium chloride (CPC) and sent by post to BRTC in Bagamoyo and processed as previously described [26].

#### Helminth investigations

For the diagnosis of helminth infections, stool and urine samples were collected once from each patient before the start of TB treatment. From the rural site, the samples were sent to the Ifakara Health Institute Helminth Laboratory in Ifakara, Tanzania. From the urban site, the samples were transferred to the Helminth Unit at BRTC in Bagamoyo. At each laboratory, samples were examined for helminth infection using standardized, quality-controlled procedures as previously described [18, 27]. The Kato-Katz method was performed in triplicate with thick stool smears from each sample to diagnose *Ascaris lumbricoides,* hookworm, *Schistosoma mansoni*, and *Trichuris trichiura* infection. *Strongyloides stercoralis* infection was diagnosed by the Baermann method [27]. Microhaematuria was examined by reagent strips (Hemastix; Siemens Healthcare Diagnostics Inc.; Tarrytown, USA). Additionally, a point-of-care circulating cathodic antigen (POC-CCA) urine cassette test (ICT Diagnostics, Noordhoek, South Africa) was employed for rapid diagnosis of *S. mansoni* [28]. *S. haematobium* eggs were detected using urine filtration [18, 29]. For quality control, 10% of the Kato-Katz slides were randomly selected and re-examined by a senior laboratory technician at each site [27].

#### Other laboratory investigations

HIV screening was done using the Alere Determine HIV rapid test (Alere; San Diego, USA) following national HIV testing algorithms; the Uni-gold HIV rapid test (Trinity Biotech; Wicklow, Ireland) served as a confirmatory test in the event of a positive screening test. In HIV-positive patients, CD4 cell counts were determined by flow cytometry (FACS Calibur, Becton Dickinson Biosciences; San Jose, USA) within 3 h after blood was drawn. A full blood cell count was done with a Sysmex XP-300 (Sysmex Corporation; Kobe, Japan). Blood tests were performed at the Ifakara Health Institute (IHI) laboratories under regular supervision by the quality assurance team.

### Data collection and definitions

At the time of recruitment, clinical officers from the Ifakara Health Institute with extensive experience in clinical research performed physical examinations and interviewed patients using standardized questionnaires [18, 22]. We collected sociodemographic, clinical, and socioeconomic data from all patients. Data were entered using tablets via the OpenDataKit application (www.opendatakit.org). Data quality was monitored in real-time using the “*odk_planner*” tool [20].

A TB patient was defined as new detection of *Mycobacterium tuberculosis* in the sputum by smear microscopy or Xpert MTB/RIF assay [24]. A new TB patient was defined as a person, who had never been treated or whose prior treatment for TB had lasted less than 1 month [28]. Recurrent TB patients (relapse patients) were persons who had been treated previously for TB and had been declared cured or had completed their most recent course of treatment, who then presented with a recurrent episode of TB (either a true relapse or a new episode of TB caused by reinfection) [28].

Severe anaemia was defined as haemoglobin (Hb) < 8.5 g/dl. Diabetes mellitus was defined as a random or fasting blood glucose level of ≥11.1 mmol/L or 7 mmol/L [30]. Patients were considered co-infected with helminths if eggs or larvae of the following species in stool or urine microscopy were present: *A. lumbricoides*, *Enterobius vermicularis*, *Hymenolepis diminuta*, hookworm, *S. haematobium*, *S. mansoni*, *S. stercoralis*, and *T. trichiura*. Additionally, *S. mansion* infection was defined as a positive POC-CCA urine cassette test. Two forms of helminth infection were distinguished: schistosomiasis, due to infection with either *S. mansoni* or *S. haematobium*; and other helminthiases, which included infection with *A. lumbricoides, E. vermicularis, H. diminuta*, hookworm, *S. stercoralis,* or *T. trichiura*. Occupational risk for acquiring helminth infection was defined as working in rice fields, car washing, or sand harvesting pits, and fishing in rivers or still bodies of natural freshwater. TB diagnosis delay was recorded whenever more than 3 weeks elapsed between occurrence of patient’s first TB symptom(s) and TB diagnosis was made [22].

### Statistical and geographical analysis

Descriptive statistics were employed for characterizing patients. For continuous variables, the Wilcoxon rank-sum or Student’s *t*-tests were used, depending on the distribution from the two sites, and *χ*^2^ or Fisher’s exact tests for comparison of categorical variables, as appropriate. We set the threshold of a statistically significant difference at an alpha level of 0.05. Univariate and multivariate logistic regression models were fitted to assess the association between recurrent TB with different epidemiological characteristics among TB patients. Additionally, we analysed the association of helminth co-infections and different predictors in a logistic regression model adjusted for age, sex, HIV infection, individual deworming history, occupational risk, and site (urban/rural). All analyses were performed in Stata version 13.1 (Stata Corporation; College Station, USA).

We collected geo-coordinates using Android tablets from the two study sites and the TB laboratory. The Tanzania regions shapefiles were obtained from the Tanzanian National Bureau of Statistics, and merged with the corresponding annual TB notification rates in 2015 obtained from the National Tuberculosis and Leprosy Programme. We used ArcGIS version 10.4 (Esri; Redlands, USA) to produce the map.

## Results

### Comparison of patient characteristics in the urban and rural setting

We studied 668 patients enrolled between August 2015 and February 2017, 460 (68.9%) at the urban and the remaining 208 (31.1%) at the rural site (Table 1). Their median age was 35 years (interquartile range [IQR]: 27–41.5 years), and 454 (68.0%) were males. Rural patients were older than those from the urban setting (median age 37 years, IQR: 27–46 years, vs. 34 years, IQR: 27–40 years; *P* = 0.003). Prevalence of HIV infection was similar in the two settings (59/208 [28.4%] in the rural vs. 108/460 [23.5%] in the urban setting, *P* = 0.18). Among HIV-positive patients from the rural setting, start of anti-retroviral treatment (ART) was delayed after HIV diagnosis compared to those from the urban setting (median time to start of ART 24 days [IQR: 11–64 days] vs. 14 days [IQR: 11.5–20.5 days], *P* = 0.001). The proportion of a contact history with a TB case was higher in rural compared to urban patients (20.2%, 95% *CI*: 14.7–25.6% vs. 12.8%, 95% *CI*: 9.8–15.9%, *P* = 0.014). Body mass index (BMI) at the time of TB diagnosis was significantly lower among rural patients than their urban counterparts (median 17.5 kg/m^2^ [IQR: 16.2–19.6 kg/m^2^] vs. 18.5 kg/m^2^ [IQR: 17–20.3 kg/m^2^], *P* <  0.001).

**Table 1:** Sociodemographic and clinical characteristics of adult tuberculosis (TB) patients at the time of diagnosis, enrolled between August 2015 and February 2017 in Dar es Salaam (urban) and Ifakara (rural), Tanzania

Characteristics	All	Urban	Rural	*P*-value
Total	668	460	208	
Age groups in years, *n* (%)				0.001
18–24	112 (16.8)	78 (17.0)	34 (16.4)	
25–33	199 (29.8)	151 (32.8)	48 (23.1)	
34–43	220 (32.9)	155 (33.7)	65 (31.3)	
≥ 44	137 (20.5)	76 (16.5)	61 (29.3)	
Sex, *n* (%)				0.81
Female	214 (32.0)	146 (31.7)	68 (32.7)	
Male	454 (68.0)	314 (68.3)	140 (67.3)	
HIV status, *n* (%)				
Negative	497 (74.4)	352 (76.5)	145 (69.7)	
Positive	167 (25.0)	108 (23.5)	59 (28.4)	0.18
Unknown	4 (0.6)	0	4 (1.9)	
Time to ART initiation in days, median (IQR)	15 (11–35)	14 (11.5–20.5)	24 (11–64)	0.001
Education level, *n* (%)				0.85
No/primary	552 (82.6)	381 (82.8)	171 (82.2)	
Secondary/university	116 (17.4)	79 (17.2)	37 (17.8)	
Occupation, *n* (%)				< 0.001
Unemployed	257 (38.5)	100 (21.7)	157 (75.5)	
Employed	411 (61.5)	360 (78.3)	51 (24.5)	
Smoking status ^a^, *n* (%)	166 (24.9)	122 (26.5)	44 (21.2)	0.18
Alcohol abuse ^b^, *n* (%)	158 (23.7)	105 (22.8)	53 (25.5)	0.46
People in the household, *n* (%)				< 0.001
≤ 3	428 (64.1)	315 (68.5)	113 (54.3)	
> 3	240 (35.9)	145 (31.5)	95 (45.7)	
History of TB contact, *n* (%)	101 (15.1)	59 (12.8)	42 (20.2)	0.014
Household monthly income, *n* (%)				0.032
< 100 USD	459 (68.7)	328 (71.3)	131 (63.0)	
≥ 100 USD	209 (31.3)	132 (28.7)	77 (37.0)	
Patient category				< 0.001
New case	643 (96.3)	454 (98.7)	189 (90.9)	
Recurrent case	24 (3.5)	6 (1.3)	18 (8.7)	
Return after lost to follow-up case	1 (0.2)	0	1 (0.5)	
BMI (kg/m^2^), median (IQR)	18.3 (16.7–20.3)	18.5 (17–20.4)	17.5 (16.2–19.6)	< 0.001
BMI categories in kg/m^2^, *n* (%)				0.001
Underweight, < 18.5	362 (54.2)	229 (49.8)	133 (63.9)	
Normal, 18.5–24.9	278 (41.6)	205 (44.6)	73 (35.1)	
Overweight, 25.0–29.9	21 (3.1)	20 (4.4)	1 (0.5)	
Obese, ≥ 30	7 (1.1)	6 (1.3)	1 (0.5)	
Hb level in g/dl, median (IQR)	11.0 (9.7–12.5)	11.1 (9.9–12.6)	11.0 (9.3–12.1)	0.27
Body fat in %, median (IQR)	10.8 (8.0–14.8)	10.6 (7.8–14.8)	11.5 (8.3–14.8)	0.087
Diagnosis delay in weeks, median (IQR)	4 (3–8)	4 (3–6)	8 (4–12)	< 0.001
Diagnosis delay in weeks, *n* (%)				< 0.001
≤ 3 weeks	174 (26.1)	153 (33.4)	21 (10.1)	
> 3 weeks	492 (73.9)	305 (66.6)	187 (89.9)	
Visiting traditional healers, *n* (%)	86 (12.9)	26 (5.7)	60 (28.9)	< 0.001
Helminth factors, *n* (%)				
Occupational risk ^c^	274 (41.0)	232 (50.4)	42 (20.2)	< 0.001
Individual deworming ^d^	377 (56.4)	304 (66.1)	73 (35.1)	< 0.001
Part of mass drug campaign ^d^	83 (12.4)	73 (15.9)	10 (4.8)	< 0.001

*ART* Antiretroviral therapy, *BMI* Body mass index, *HIV* Human immunodeficiency virus, *Hb* Haemoglobin level, *IQR* Inter-quartile range

*USD* United States Dollars (1 USD = 2171 Tanzanian Shillings, June 2016)

^a^Defined as current smoking

^b^Alcohol use defined as drinking alcohol regularly—at least three standard bottles of beer (or equivalent) per day

^c^Occupational risk for helminth infection defined as working in rice fields, car washing, harvesting sand, or fishing

^d^Deworming practice in past 12 months

At the time of TB diagnosis, mean Hb levels were comparable in both groups. Among HIV-positive TB patients, the median CD4 cell count was lower in the rural compared to the urban setting (147 cells/μl [IQR: 84–246 cells/μl] vs. 249 CD4 cells/μl [IQR 131–450 cells/μl], *P =* 0.02). Among 630 patients whose blood glucose levels were tested, 17/630 (2.5%) were diagnosed as diabetic. Prevalence of diabetes in the TB patients did not differ between the two settings.

TB patients in the rural setting were less likely to self-access anthelminthic medications than urban patients (73/208 [35.1%] vs. 304/460 [66.1%], *P* <  0.001) and had less access to mass deworming campaigns in the last 12 months at the time of recruitment (10/208 [4.8%] vs. 73/460 [15.9%], *P* <  0.001).

### Comparison of recurrent TB cases in the urban and rural setting

In the rural setting, 18/208 (10.0%) were recurrent (relapse) TB cases compared to 6/460 (1.3%) in the urban setting. Patients with recurrent TB were older than patients with a first TB episode (47 years vs. 35 years), 22 of the 24 patients with recurrent TB were males, and 14 were not employed. Rural recurrent TB patients had a higher median body fat percentage than those from the urban setting (11.9% vs. 7.1%, *P* = 0.005). Characteristics of recurrent TB patients are described in Table 2.

**Table 2:** Characteristics of recurrent TB cases in urban and rural Tanzania. Patients enrolled between August 2015 and February 2017 in Dar es Salaam (urban) and Ifakara (rural)

Characteristics	All	Urban	Rural	*P*-value
Total, *n* (%)	24 (100)	6 (25.0)	18 (75.0)	< 0.001
Age in years, median (IQR)	47 (33.5–53)	39 (29–47)	47.5 (38–55)	0.20
Male sex, *n* (%)	22 (91.7)	6 (100)	16 (88.9)	0.55
HIV infection, *n* (%)	5 (20.8)	1 (16.7)	4 (22.2)	1.0
Education level, *n* (%)	5 (0.9)			0.55
No/primary school	21 (87.5)	15 (83.3)	6 (100)	
Secondary/university	3 (12.5)	3 (16.7)	–	
Occupation, *n* (%)				0.002
Unemployed	14 (58.3)	–	14 (77.8)	
Employed	10 (41.7)	6 (100)	4 (22.2)	
Smoking ^a^, *n* (%)	3 (12.5)	2 (33.3)	1 (5.6)	0.25
Alcohol use ^b^, *n* (%)	4 (16.7)	–	4 (22.2)	0.54
Diabetes mellitus	1 (5.3)	–	1 (7.7)	–
People in the household, *n* (%)				0.99
≤ 3	17 (70.8)	4 (66.7)	13 (72.2)	
> 3	7 (29.2)	2 (33.3)	5 (27.8)	
History of TB contact, *n* (%)	7 (29.2)	1 (16.7)	6 (33.3)	0.63
Household monthly income, *n* (%)				0.99
< 100 USD	18 (75.0)	5 (83.3)	13 (72.2)	
≥ 100 USD	6 (25.0)	1 (16.7)	5 (27.8)	
BMI in kg/m^2^, median (IQR)	16.9 (16.3–18.4)	16.5 (15.7–17.0)	17.1 (16.5–18.8)	0.14
Body fat in %, median (IQR)	10.9 (7.4–14.9)	7.1 (5.9–8.5)	11.9 (10.2–15.2)	0.005
Hb level in g/dl, median (IQR)	10.8 (10–12)	11.2 (7.2–12.9)	10.5 (10.1–12)	0.79
Visiting traditional healers, *n* (%)	5 (20.8)	1 (16.7)	4 (22.2)	0.99
Previous use of antibiotics, *n* (%)	18 (75.0)	3 (50.0)	15 (83.3)	0.14
Any helminth infection, *n* (%)	5 (22.7)	2 (50.0)	3 (16.7)	0.21

*n* Number, *IQR* Interquartile range, *HIV* Human immunodeficiency virus, *USD* United States Dollars (1 USD = 2171 Tanzanian Shillings, June 2016)

^a^Defined as current smoking

^b^Alcohol use defined as drinking alcohol regularly—at least three standard bottles of beer (or equivalent) per day

In a multivariate logistic regression model, we found that rural patients were more likely to have recurrent TB than urban patients (adjusted odds ratio [a*OR*]: 3.97, 95% *CI*: 1.16–13.67, *P* = 0.029). Furthermore, for each 1-year increase in age, the adult TB patient risk of developing recurrent TB increased by 6% (a*OR*: 1.06, 95% *CI*: 1.02–1.10, *P* = 0.001). We found that patients who were underweight (BMI <  18.5 kg/m^2^) had a higher risk of recurrent TB (a*OR*: 2.97, 95% *CI*: 0.85–10.30, *P* = 0.087; Table 3).

**Table 3:** Factors associated with recurrent tuberculosis (TB) among TB cases in urban and rural Tanzania

Characteristics	Crude			Adjusted		
*n* (%)	*OR*	(95% *CI*)	*P*-value	*OR*	(95% *CI*)	*P*-value
Demographics						
Age (years)	1.06	(1.04–1.09)	< 0.001	1.06	(1.02–1.10)	0.001
Male sex	4.42	(1.18–16.5)	0.027	3.04	(0.73–12.6)	0.13
BMI < 18.5 kg/m^2^	2.47	(0.99–6.11)	0.051	3.0	(0.85–10.3))	0.087
Body fat (%)	0.98	(0.91–1.04)	0.50	–	–	–
*Social* characteristics						
Higher education level ^a^	0.67	(0.2–2.29)	0.52	–	–	–
Employed	0.44	(0.2–0.99)	0.046	1.52	(0.47–4.87)	0.49
Monthly income > 200 USD	0.76	(0.31–1.89)	0.56	–	–	–
Living in the rural	6.79	(2.73–16.86)	< 0.001	3.97	(1.16–13.67)	0.029
Household members > 3	0.76	(0.32–1.8)	0.53	–	–	–
History of TB contact	2.5	(1.03–6.04)	0.04	1.66	0.55–5.03	0.37
Individual deworming ^b^	0.38	(0.17–0.89)	0.026	0.59	(0.19–1.76)	0.34
Occupational risk ^c^	1.04	(0.46–2.34)	0.92	–	–	–
Smoking	0.48	(0.15–1.50)	0.21	–	–	–
Alcohol abuse	0.70	(0.25–1.96)	0.50	–	–	–
Comorbidities						
HIV infection	1.38	(0.66–2.88)	0.39	0.51	(0.19–1.38)	0.13
Diabetes mellitus	2.93	(0.52–16.60)	0.23	3.25	(0.43–4.65)	0.25
Severe anaemia ^d^, g/dl	1.07	(0.34–3.37)	0.91	–	–	–
Haematuria	0.3	(0.02–5.10)	0.41	–	–	–
Any helminth infection	0.99	(0.38–2.65)	1.0	–	–	–

*OR* Odds ratio, *CI* Confidence interval, *HIV* Human immunodeficiency virus, *BMI* Body mass index

Logistic regression model adjusted for age, sex, BMI, employment status, setting (urban/rural), history of TB contact in the household, individual deworming, HIV infection and diabetes

^a^Higher education level consists of TB patients who completed their secondary of university education

^b^Individual deworming habit in the last 12 months prior to TB diagnosis

^c^Occupational risk for helminth infection defined as working in rice fields, car wash, harvesting sand, or fishing

^d^Severe anaemia defined as blood haemoglobin (Hb) level < 8.5 g/dl

### Comparison of helminth co-infections patterns and associated risk factors in the urban and rural settings

The overall prevalence of helminth co-infections in TB patients was 23.1%. As shown in Table 4, the prevalence of helminth co-infections was significantly higher in the urban compared to the rural setting (25.7%, 95% *CI*: 21.7–29.6% vs. 17.3%, 95% *CI*: 12.2–22.5%; *P* = 0.02). The rural setting had a higher prevalence of *S. mansoni* than the urban setting and a lower prevalence of *S. stercoralis*. Figure 3 shows the distinctive pattern of helminth co-infections.

**Table 4:** Frequency distribution of helminth infection among adult TB patients in Dar es Salaam (urban) and Ifakara (rural), Tanzania

Helminth infection	All, *n* (%)	Urban		Rural		*P*-value
		*n* (%)	95% *CI*	*n* (%)	95% *CI*	
Total	668 (100)	460 (68.9)		208 (31.1)		
Any helminth infection ^a^	154 (23.1)	118 (25.7)	21.7–29.6	36 (17.3)	12.2–22.5	0.018
Soil-transmitted helminths infections ^b^						
*Strongyloides stercoralis*	89 (13.3)	79 (17.2)	14.4–21.5	10 (4.8)	1.9–7.8	< 0.001
Hookworm	45 (6.7)	26 (5.7)	3.7–8.1	19 (9.1)	5.3–13.2	0.12
*Ascaris lumbricoides*	2 (0.3)	–	–	2 (1.0)	–	–
*Trichuris trichiura*	5 (0.8)	2 (0.4)	–	3 (1.4)	–	–
Schistosomiasis						
*Schistosoma mansoni* ^b^	15 (2.3)	8 (1.7)	0.57–3.1	7 (3.4)	0.9 to 5.9	0.21
*Schistosoma mansoni* ^c^	53 (7.9)	19 (4.13)	2.4–6.2	34 (16.4)	11.4–21.6	< 0.001
1+	19 (43.2)	0	–	19 (63.3)	–	–
2+	6 (13.6)	0	–	6 (20.0)	–	–
3+	19 (43.2)	14 (100)		5 (16.7)	–	–
*Schistosoma haematobium* ^d^	19 (2.8)	16 (3.5)	1.9–5.4	3 (1.4)	–	–
Multiple helminth infection						0.63
None	471 (70.5)	322 (70.0)	65.8–74.2	149 (71.6)	65.5–77.7	
Mono-infection	159 (23.8)	108 (23.5)	19.6–27.4	51 (24.5)	18.7–30.3	
Infection with ≥2 species	16 (2.4)	10 (2.2)	0.86–3.5	6 (2.9)	0.62–5.2	

*TB* Tuberculosis, *POC-CCA* Point-of-care circulating cathodic antigen

^a^Including POC-CCA positive tests (*Schistosoma mansoni*)

^b^Based on stool microscopy

^c^Based on POC-CCA test only

^d^Based on urine filtration

In a multivariate analysis, TB patients from the urban setting had significantly higher odds of having any helminth infection at the time of TB diagnosis compared to those from the rural setting (a*OR*: 2.44, 95% *CI*: 1.44–4.14, *P* = 0.001). TB patients who had taken anthelminthic medication within 12 months prior to TB diagnosis had lower odds of having acquired a helminth infection (a*OR*: 0.59, 95% *CI*: 0.39–0.88, *P* = 0.009). Neither age, sex, HIV infection, occupational risk, nor any other cofactor was associated with helminth infection.

## Discussion

Among the 668 TB patients from the urban and rural settings in Tanzania we studied, patients from the rural setting were older, had a lower average BMI, and lower median CD4 cell counts in case of HIV co-infection. Moreover, patients from the rural setting had more frequently recurrent TB. Whereas schistosomiasis was higher in the rural setting, the overall prevalence of helminth infections was higher in the urban setting, especially due to *S. stercoralis*.

We found differences in patient characteristics between urban and rural settings, such as age, BMI, occupation, as well as health seeking behavior (e.g., use of antibiotics prior to TB diagnosis and consultation of traditional healers) as reported previously from rural Tanzania [31]. The difference in time between HIV diagnosis and ART initiation in the two sites is likely explained by greater distances of rural patients to the treatment center, a factor that has been shown to contribute in treatment delay [31, 32]. Rural patients comparatively had increased likelihood of visiting traditional healers due to poorer access to health facilities [33]. The resulting delay in seeking medical care has been shown to contribute to ongoing TB transmission [5, 7, 31, 34].

Helminth co-infections have been associated with rural residence [35, 36]. In our recent work in Tanzania, we also found a positive association between TB and helminth co-infections in an urban setting [18]. The observed higher prevalence of helminths in the urban setting could be due to the rapid growth of the city with poor urban planning and hygiene control. This results in slum-like dwellings in large parts of the city with higher risk of infection with helminths [37]. The overall prevalence of TB and helminth co-infections we observed was comparable to previous studies elsewhere in sub-Saharan Africa [18, 36, 38–41]. The prevalence of *S. stercoralis*, the principal driver of helminthiasis in the urban setting in the current study, was comparable to that seen in previous investigations from urban Tanzania and rural Ghana [18, 42]. The high prevalence of schistosomiasis we observed in the rural setting is likely to be setting-specific, with Ifakara Town being close to the Kilombero River. This was also shown in rural areas of the Democratic Republic of the Congo [43], especially among those who came in regular contact with natural open freshwater bodies (e.g., fishermen and rice famers) [44, 45]. Environmental conditions and administration of anthelminthic medication initiated on an individual basis or during mass deworming campaigns differed across our two settings, and these two factors affect prevalence and patterns of helminth co-infections. When designing public health interventions, such differences must be taken into consideration for further improvement of clinical outcomes in TB patients, as helminth co-infections alter clinical presentation and immune response to infection [18, 38, 39, 41].

The association of recurrent TB with older age and living in certain areas has also been observed elsewhere [46, 47], and particular comorbidities, such as HIV and diabetes mellitus, have been associated with recurrent TB [46, 48, 49]. Recurrent TB is more common in HIV-positive than HIV-negative patients [46, 50, 51], and is related to poor treatment outcomes [46]. Smoking has been associated with a three-fold increased risk of developing recurrent TB in India [52]. However, this finding could not be confirmed in the present study. Because most recurrent cases of TB occur within 12 months of completion of treatment, follow-up after completion of treatment is important [52, 53].

A limitation of our study was its inability to differentiate reinfection from relapse among recurrent TB cases because we did not have sputum samples from prior episodes that would have made possible differentiation by molecular genotyping of *M. tuberculosis* isolates. Drug resistance information was also lacking, except for rifampicin resistance tested by Xpert MTB/RIF assay. Although multiple techniques were used for identification of helminth co-infections, the investigation of a single stool sample in our study could have resulted in underestimation of the true prevalence of infection, especially of hookworm, *T. trichiura* and *A. lumbricoides* [54].

## Conclusions

The differences in clinical and socio-demographic characteristics of TB patients in urban and rural Tanzania underline that public health interventions need to be tailored to a given setting to improve clinical outcomes of TB and mitigate the risk of co-infections. TB patients in the rural Tanzania are likely to be older with more recurrent TB cases, have more limited access to anthelminthic medication individually, have a longer TB diagnosis delay, and seek more frequently care from traditional healers. The overall prevalence of helminth co-infections in TB patients was higher in the urban setting, predominantly driven by *S. stercoralis* infection, but the prevalence of *S. mansoni* was higher in the rural setting. These observations may guide public health interventions that target, for example, traditional healers in the rural setting, aiming to improve early detection of TB cases and referral for anti-tuberculosis treatment. On the other hand, screening and treatment of helminths among TB patients should be improved, especially in the urban setting.

## Additional file

Additional file 1: Multilingual abstracts in the five official working languages of the United Nations. (PDF 696 kb)

## Abbreviations

a*OR*: Adjusted odds ratio
ART: Anti-retroviral therapy
BMI: Body mass index
BRTC: Bagamoyo Research and Training Center
CDCI: Chronic Diseases Clinic of Ifakara
CPC: Cetylpyridinium chloride
Hb: Haemoglobin
HIV: Human immune deficiency virus
IQR: Inter quartile range
LED: Light emitting diode
OR: Odds ratio
POC-CCA: Point-of-care circulating cathodic antigen
TB: Tuberculosis
USD: United States dollar
