# Supplementary material for: Distinct clinical characteristics and helminth co-infections in adult tuberculosis patients from urban compared to rural Tanzania
Source: Infect Dis Poverty. 2018 Mar 24;7:24. doi: 10.1186/s40249-018-0404-9 (PMC5868052; doi:10.1186/s40249-018-0404-9)
Supplement: Supplementary file 1 — Multilingual abstracts in the five official working languages of the United Nations. (PDF 696 kb) [file 40249_2018_404_MOESM1_ESM.pdf]

Translation of the abstract into the six official working languages of the United Nations

الخصائص السريرية المتباينة والعدوى بالديدان الطفيلية في مرضى السل البالغين من المناطق الحضرية في تنزانيا مقارنة مع المناطق الريفية فيها.

جورج سيكالنغو، جيري هيلا، فرانسيس مهيمبيرا، ليليانا ك. روتايهوا، فريدة بَني، روبرت نديج، محمد ساسامالو، لوجيكو كامويلا، خديجة سعيد، جريس مالو، يورومين ملشا، كريستوف هاتس، ستيفاني نوب، سيباستيان غانيوكس، كلاوس ريثر، يورغ أوتزينجر، مارسيل تانر، إميليو ليتانغ، ماجا فايسر، لوكاس فينر

الملخص

خلفية: إن الاختلافات في الظروف القروية والمدنية يمكن أن تفسر الخصائص المتباينة في الدراسة الوبائية لمرض السل درسنا بشكل مقارنة نسبية الصفات الوبائية لمرض السل وعدوى الديدان الطفيلية في المرضى البالغين ضمن شروط المناطق القروية والحضرية في تانزانيا

تم تسجيل مرضى البالغين (≤ 18 سنة) المصابين بالسل الرئوي المؤكدة ميكروبيولوجيا على التوالي في مجموعتين في دار السلام، ويبلغ عدد سكانها 4،4 مليون نسمة (في المناطق الحضرية)، وإيفاكارا في منطقة كيلومبيرو ذات الكثافة السكانية المنخفضة التي يبلغ عدد سكانها حاليا 400،000 نسمة (في المناطق الريفية). تم الحصول على البيانات السريرية عند التطويع. وتعرضت عينات البراز والبول لتشخيص الديدان الطفيلية باستخدام كاتو كاتس وبيرمان وترشيح البول وتجارب أدوية المناعة الكاثودية. تُقدر الاختلافات بين المجموعات ب X^{-2-} اختبارات فيتشر *للدقة، واختبار يلكوكسون لجمع القيم استخدمت نماذج التراجع اللوجستي لتحديد الروابط.*

النتائج: بين آب / أغسطس 2015 وشباط / فبراير 2017، التحق 668 مريضاً، و 460 (68.9٪) في المناطق الحضرية و 208 (31.1٪) في الموقع الريفي. وكان متوسط عمر المريض 35 عاماً (المدى بين الربعين: 27-41.5 سنة)، و 454 (68٪) من الذكور. المرضى في الشروط القروية كانوا أكبر(كان متوسط العمر 37 سنة بالمقابل) 34 عام (النسبة =0.003), لديهم مؤشر متوسط كتلة جسم منخفضة أكثر(17.5 كجم/م$^{-2}$-مقابل 18.5 كجم/م$^{-2}$-, بنسبة < 0.001)، ونسب أعلى لحالات مرض السل المتكررة(9% مقابل 1% بنسبة <0.001)، ومتوسط تعداد خلايا منخفض أكثر في كتلة التمايز4 لدى المرضى المصابين بمرض الإيدز والسل سوياً(147 خلية/ميكرولتر بالمقابل 249 خلية/ميكرولتر بنسبة =0.02) مقارنة بالمرضى من تانزانيا الحضرية. لم يكن هناك فرق واضح في تكرار مرض الإيدز، داء السكري، نسب تركيز خضاب الدم بين شروط البيئيتين. إن نسبة الانتشار العام لعدوى الطفيليات 22.9% (95% مرحلة الثقة:(20.4 - 27.0%) إن الانتشار الكبير لعدوى الديدان الطفيلية في المواقع الحضرية (25.7% . مقابل 17.3%، بنسبة = 0.018)، يرجع في الغالب إلى الدودة الخيطية (17.0% 4.8%, بنسبة< 0.00)، وعدوى البلهارسية المنسونية (4.1% مقابل 16.4%, بنسبة < 0.001) كانت عدوى مرض السل مترافقة مع العيش في الشروط الريفية (نسبة الأرجحية المُعدلة 3.97, 95% CI: 1.16–13.67)، والعمر المتزايد (ORa: 1.06, 95% CI: 1.02–1.10)

<خاتمة: إن الخصائص السريرية ونماذج عدوى الطفيليات تختلف في مرضى السل في تانزانيا القروية عنها في المناطق الحضرية. وتؤكد الفروق على الحاجة إلى تدخلات محددة في الصحة العامة مصممة خصيصاً لتحسين التدبير السريري لمرض السل والأمراض المصاحبة.

Translated from English version into Arabic by Dima shs, proofread by Dr. Manale Elewah, through

TRANSLATORS
WITHOUT BORDERS

与农村地区相比，坦桑尼亚城市成人结核病患者具有明显的临床特征和蠕虫合并感染

George Sikalengo, Jerry Hella, Francis Mhimbira, Liliana K. Rutaihwa, Farida Bani, Robert Ndege, Mohamed Sasamalo, Lujeko Kamwela, Khadija Said, Grace Mhalu, Yeromin Mlacha, Christoph Hatz,

Stefanie Knopp, Sébastien Gagneux, Klaus Reither, Jürg Utzinger, Marcel Tanner, Emilio Letang, Maja Weisser and Lukas Fenner

摘要

引言：农村和城市环境的差异能够解释结核病的流行病学特征。我们对坦桑尼亚农村和城市成年结核病患者及蠕虫合并感染的流行病学特征进行了比较研究。

方法：研究人员分别在人口约为 440 万的达累斯萨拉姆（城市），和人口约为 40 万的 Kilombero 地区的 Ifakara（农村）招募两个队列研究。成年患者(≥18 岁)通过微生物学确诊为肺结核，并在招募时获取临床资料。使用改良加藤法、Baermann、尿液过滤和循环阴极抗原检测粪样和尿样，以诊断蠕虫感染。通过 χ^2 检验、费舍尔确切概率、Wilcoxon 等级评估组间差异，使用 Logistic 回归模型来确定关联关系。

结果：2015 年 8 月至 2017 年 2 月期间，共有 668 名患者入选，其中，460 例(68.9%)在城市，208 例(31.1%)在农村。患者年龄中位数为 35 岁[四分位间距(*IQR*): 27–41.5 岁]，男性 454 例(68%)。与坦桑尼亚城市患者相比，农村患者年龄较大(平均年龄 37 *vs.* 34，$P = 0.003$)、中位体重指数较低（17.5 kg/m^2 *vs.* 18.5 kg / m^2，$P < 0.001$）、复发性结核病例比例较高（9% *vs.* 1%, $P < 0.001$）。艾滋病/结核病合并感染患者的中位数 CD4 细胞计数较低（147 细胞/μl *vs.* 249 细胞/μl，$P = 0.02$）。然而 HIV 感染和糖尿病的发生率，以及血红蛋白浓度，两组间的差异无统计学意义。蠕虫和结核病合并感染的总体患病率为 22.9% [95%置信区间（*CI*）: 20.4-27.0%]。在城市地区，蠕虫感染患病率明显较高（25.7% *vs.* 17.3%，$P = 0.018$），主要是粪类圆线虫（17.0% *vs.* 4.8%，$P < 0.001$）和曼氏血吸虫感染（4.1% *vs.* 16.4%，$P < 0.001$）所致。复发性结核与患者生活在农村 [调整优势比（a*OR*）: 3.97, 95% *CI*: 1.16–13.67]）和年龄增长（a*OR*: 1.06, 95% *CI*: 1.02–1.10）有关。

结论：坦桑尼亚城市和农村地区，结核病患者的临床特征和蠕虫合并感染模式不同。这些差异强调了需要制定对应的公共卫生干预措施以改善结核病和合并感染的临床管理。

Translated from English version into Chinese by Translated by Xin-Yu Feng, edited by Pin Yang

CHINESE CENTER FOR DISEASE CONTROL AND PREVENTION
NATIONAL INSTITUTE OF PARASITIC DISEASES

Comparaison des caractéristiques cliniques distinctes et co-infections par les helminthes chez les patients adultes atteints de tuberculose vivant en Tanzanie rurale et urbaine

George Sikalengo, Jerry Hella, Francis Mhimbira, Liliana K. Rutaihwa, Farida Bani, Robert Ndege, Mohamed Sasamalo, Lujeko Kamwela, Khadija Said, Grace Mhalu, Yeromin Mlacha, Christoph Hatz, Stefanie Knopp, Sébastien Gagneux, Klaus Reither, Jürg Utzinger, Marcel Tanner, Emilio Letang, Maja Weisser et Lukas Fenner

Résumé

Contexte: les différences entre les zones urbaines et rurales pourraient expliquer les caractéristiques distinctes de l'épidémiologie de la tuberculose (TB). Nous avons procédé à une étude comparative des caractéristiques épidémiologiques de la TB et des co-infections dues aux helminthes chez les patients adultes vivant dans des zones urbaines et rurales de Tanzanie.

Méthodologie: des patients adultes (≥ 18 ans) atteints de TB pulmonaire confirmée par microbiologie ont été inclus dans deux cohortes à Dar es Salaam (zone urbaine, ≈ 4,4 millions d'habitants) et à Ifakara,

dans le district à la population éparse de Kilombero (zone rurale, ≈ 400 000 habitants). Des données cliniques ont été recueillies au moment de leur inclusion. Des tests d'antigène cathodique circulant, de filtration d'urine, de Baermann et de Kato-Katz ont été employés sur des échantillons de selles et d'urine afin de diagnostiquer toute helminthiase. Les différences entre les groupes ont été mesurées à l'aide du test du χ^2, du test exact de Fisher et du test de somme des rangs de Wilcoxon. Des modèles de régression logistique ont permis de déterminer les associations.
Résultats: entre août 2015 et février 2017, 668 patients ont été inclus, 460 (68,9 %) en zone urbaine et 208 (31,1 %) en zone rurale. L'âge médian des patients était de 35 ans (intervalle interquartile : 27 à 41,5 ans). 454 patients (68 %) étaient des hommes. Les patients en zone rurale étaient plus âgés (âge médian : 37 ans *contre* 34, $P = 0,003$), avaient un indice de masse corporelle médian inférieur (17,5 kg/m^2 *contre* 18,5 kg/m^2, $P < 0,001$), une plus grande proportion de cas chroniques de TB (9 % *contre* 1 %, $P < 0,001$) et, pour les patients souffrant de co-infection VIH/TB, un nombre de cellules CD4 médian inférieur (147 cellules/µl *contre* 249 cellules/µl, $P = 0,02$) en comparaison avec des patients vivant en Tanzanie urbaine. Il n'y a pas eu de différence significative dans la fréquence des infections par le VIH, des cas de diabète sucré et dans les niveaux de concentration d'hémoglobine entre les patients des deux zones. La prévalence globale des co-infections par les helminthes s'élevait à 22,9 % (intervalle de confiance à 95 % [*IC*] : 20,4 à 27,0 %). La prévalence des infections par les helminthes significativement plus élevée en zone urbaine (25,7 % *contre* 17,3 %, $P = 0,018$) était principalement le fait d'infections par *Strongyloides stercoralis* (17,0 % *contre* 4,8 %, $P < 0,001$) et *Schistosoma mansoni* (4,1 % *contre* 16,4 %, $P < 0,001$). Les cas de TB chroniques ont été associés à la vie en zone rurale (rapport de cotes corrigé [*RC*c] : 3,97, *IC*à 95 % : 1,16 à 13,67) et à l'âge plus avancé (*RC*c : 1,06, *IC*à 95 % : 1,02 à 1,10).
Conclusions: les caractéristiques cliniques et le modèle des co-infections par les helminthes diffèrent chez les patients atteints de tuberculose en Tanzanie urbaine et rurale. Ces différences mettent en exergue la nécessité d'interventions de santé publique adaptées à la zone touchée dans l'optique d'améliorer la prise en charge clinique de la TB et de ses comorbidités.

Translated from English version into French by Frank and Veronique Haour, through

Отличительные клинические характеристики и гельминтозное коинфицирование взрослых пациентов, страдающих туберкулёзом, в городских районах Танзании в сравнении с сельскими районами государства

Джордж Сикаленго, Джерри Хелла, Фрэнсис Мхимбира, Лилиана К. Рутаихва, Фарида Бани, Роберт Ндеге, Мохамед Сасамало, Лужеко Камвела, Хадия Саид, Грейс Мхалу, Иероним Млача, Кристоф Хатц, Стефани Нопп, Себастиен Гажню, Клаус Рейтер, Юрг Утцингер, Марсель Тэннер, Эмилио Летанг, Майя Вайссер и Люкас Феннер

Аннотация

История вопроса: Различия между сельскими и городскими районами могут объяснять отличительные особенности эпидемиологии туберкулёза (ТБ). Мы провели сравнительный анализ эпидемиологических характеристик ТБ и гельминтозных коинфекций у взрослых пациентов из сельских и городских районов Танзании.

Методы: Взрослые пациенты (≥ 18 лет) с микробиологически подтверждённым туберкулёзом лёгких были последовательно зачислены в две группы: в Дар-эс-Саламе, с населением около 4,4 млн человек (город), и в Ифакаре, в малонаселенном регионе Киломберо, с населением около 400 тыс. человек (сельский район). Клинические данные были собраны во время набора участников. Образцы стула и мочи были протестированы на гельминтозы с помощью метода Като-Катца, Бермана, фильтрации мочи и тестов на циркулирующий катодный антиген. Разница между группами была оценена на основании χ^2, точного теста Фишера и критерия Уилкоксона. Для определения связей были использованы модели логистической регрессии.

Результаты: В период с августа 2015 г. до февраля 2017 г. в исследовании приняли участие 668 пациентов: 460 (68,9%) в городском районе и 208 (31,1%) - в сельском. Срединный возраст пациентов составлял 35 лет (межквартильный размах: 27-41,5 лет), и 454 участников (68%) были мужского пола. Пациенты из сельского района были старше (срединный возраст - 37 лет *в сравнении с* 34 годами, $P = 0{,}003$), имели более низкое срединное значение индекса массы тела (17,5 кг/м^2 *в сравнении с* 18,5 кг/м^2, $P < 0{,}001$), более высокое соотношение случаев рецидивирующего туберкулеза (9% *в сравнении с* 1%, $P < 0{,}001$), а у пациентов с коинфекцией ВИЧ/ТБ наблюдалось более низкое срединное значение числа CD4-клеток (147 клеток/µl *в сравнении с* 249 клеток/µl, $P = 0{,}02$) у группы из городского района Танзании. Между двумя районами не было обнаружено больших различий в отношении частоты ВИЧ-инфицирования, сахарного диабета и уровней концентрации гемоглобина. Общее распространение гельминтозных коинфекций составляло 22,9% (95% доверительный интервал [*CI*]: 20,4–27,0%). Значительно более высокое распространение гельминтозного инфицирования в городском районе (25,7% *в сравнении с* 17,3%, $P = 0{,}018$) было преимущественно обусловлено *инфекциями Strongyloides stercoralis* (17,0% *в сравнении с* 4,8%, $P < 0{,}001$) и *Schistosoma mansoni* (4,1% *в сравнении с* 16,4%, $P < 0{,}001$). Случаи рецидивирующего туберкулёза были связаны с проживанием в сельской местности (скорректирование отношение шансов [a*OR*]: 3,97, 95% *CI*: 1,16–13,67) и пожилым возрастом (a*OR*: 1,06, 95% *CI*: 1,02–1,10).

Заключение: Клинические характеристики и тенденции гельминтозного коинфицирования различаются пациентов, страдающих туберкулёзом, в городских и сельских районах Танзании. Эти различия подчёркивают необходимость разработки эффективных мер общественного здравоохраненния, для конкретного района, с целью улучшения клинической работы с туберкулёзом и сопутствующими заболеваниями.

Translated from English version into Russian by Liudmila Tomanek and Elena McDonnell, through

TRANSLATORS
WITHOUT BORDERS

Comparación de las características clínicas distintivas y la infección conjunta por helmintos entre pacientes adultos con tuberculosis de las zonas urbanas y de las zonas rurales de Tanzania.

George Sikalengo, Jerry Hella, Francis Mhimbira, Liliana K. Rutaihwa, Farida Bani, Robert Ndege, Mohamed Sasamalo, Lujeko Kamwela, Khadija Said, Grace Mhalu, Yeromin Mlacha, Christoph Hatz, Stefanie Knopp, Sébastien Gagneux, Klaus Reither, Jürg Utzinger, Marcel Tanner, Emilio Letang, Maja Weisser y Lukas Fenner

Resumen

Antecedentes: Las diferencias entre el marco rural y el urbano podrían explicar las características distintivas de la epidemiología de la tuberculosis (TB). Llevamos a cabo un estudio comparativo de las características epidemiológicas de la TB y la infección conjunta por helmintos entre pacientes adultos de zonas rurales y urbanas de Tanzania.

Metodología: Se inscribieron de forma consecutiva pacientes adultos (≥ 18 años) con confirmación microbiológica de TB pulmonar, en dos grupos; uno en Dar es Salaam, con alrededor de 4,4 millones de habitantes (urbano), y otro en Ifakara, en el poco poblado Distrito Kilombero, con alrededor de 400.000 habitantes (rural). Se obtuvieron los datos clínicos en el momento del reclutamiento. Se utilizaron las técnicas de Kato-Katz, Baermann, filtración de orina y pruebas de antígenos catódicos circulantes, en muestras de materia fecal y orina para diagnosticar helmintiasis. Las diferencias entre los grupos se evaluaron con χ^2, la prueba exacta de Fisher y la prueba de los rangos con signo de Wilcoxon. Se utilizaron modelos de regresión logística para determinar las asociaciones.

Resultados: Entre agosto de 2015 y febrero de 2017, se inscribieron 668 pacientes: 460 (68,9%) en la zona urbana y 208 (31,1%) en la rural. La edad promedio de los pacientes era de 35 años (rango intercuartílico [IQR]: 27-41,5 años), y 454 (68%) de los participantes eran varones. Los pacientes de la ubicación rural eran de mayor edad (edad promedio: 37 años *frente a* 34 años, $P = 0{,}003$), tenían una menor mediana de índice de masa corporal (17,5 kg/m^2 *frente a* 18,5 kg/m^2; $P < 0{,}001$), y una mayor proporción de casos de TB recurrente (9% *frente a* 1%, $P < 0{,}001$). Además, los pacientes con infección conjunta de VIH y TB había una menor mediana de recuento de células CD4 (147 células/µl *frente a* 249 células/µl, $P = 0{,}02$) en comparación con los de la zona urbana de Tanzania. No se observaron diferencias significativas en las frecuencias de infección por VIH, ni de diabetes mellitus, ni en los niveles de concentración de hemoglobina entre los dos escenarios. La prevalencia general de las infecciones conjuntas por helmintos fue de 22,9% (intervalo de confianza del 95% [*CI*]: 20,4–27,0%). La prevalencia mucho mayor de infecciones por helmintos en el escenario urbano (25,7% *frente a* 17,3%, $P = 0{,}018$) se debe principalmente a la infección con *Strongyloides stercoralis* (17,0% *frente a* 4,8%, $P < 0{,}001$) y con *Schistosoma mansoni* (4,1% *frente a* 16,4%, $P < 0{,}001$). Se asoció la TB recurrente con el hecho de vivir en una zona rural (proporción de probabilidades ajustadas [a*OR*]: 3,97; 95% *CI*: 1,16–13,67) y con el incremento de edad (a*OR*: 1,06; 95% *CI*: 1,02–1,10).

Conclusiones: Las características clínicas y los patrones de infecciones conjuntas por helmintos de los pacientes con TB de las zonas urbanas de Tanzania difieren de los de los pacientes con TB de las zonas rurales. Estas diferencias recalcan la necesidad de intervenciones en materia de salud pública que se adopten a entornos concretos para mejorar el manejo clínico de la TB y las comorbilidades.

Translated from English version into Spanish by Camila Kohen and María Paula Gorgone, through

TRANSLATORS
WITHOUT BORDERS
